# Supplementary material for: Self-organized centripetal movement of corneal epithelium in the absence of external cues
Source: Nat Commun. 2016 Aug 8;7:12388. doi: 10.1038/ncomms12388 (PMC4979063; doi:10.1038/ncomms12388)
Supplement: Supplementary Information — Supplementary Figures 1-8, Supplementary Tables 1-2, Supplementary Methods and Supplementary References [file ncomms12388-s1.pdf]

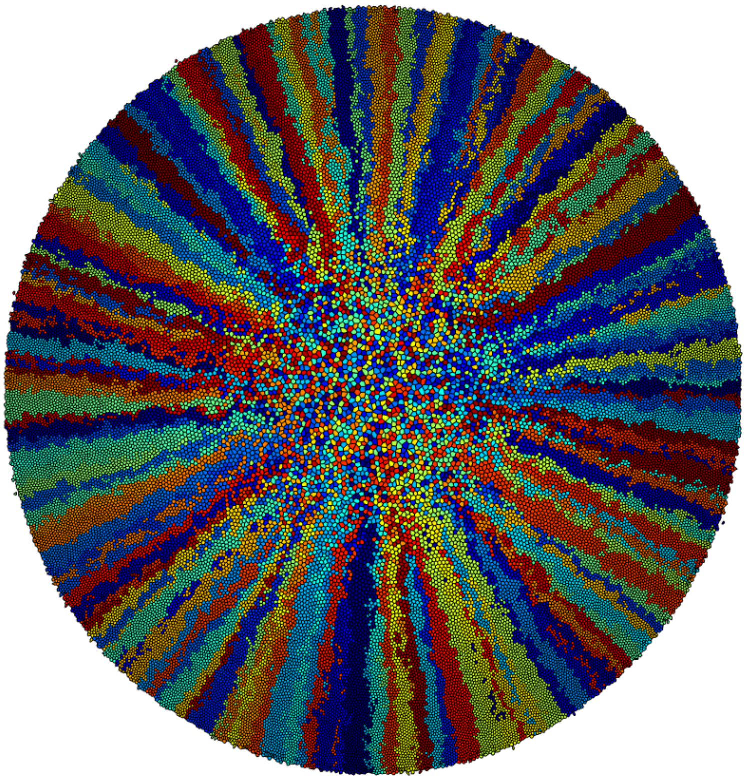

Supplementary Figure 1. **Mathematical simulation of a life-size mouse cornea maintains centripetal migration.** The radius of the simulated cornea was adjusted to be 100 cells, the radius of a mouse cornea[1]. The outcome of 2,000 time-steps, demonstrating clearly centripetal migration, is shown. A tendency for the model to produce a larger cell size in the center of the cornea, due to an incomplete pressure equilibration, is exaggerated by the large overall size of the cornea.

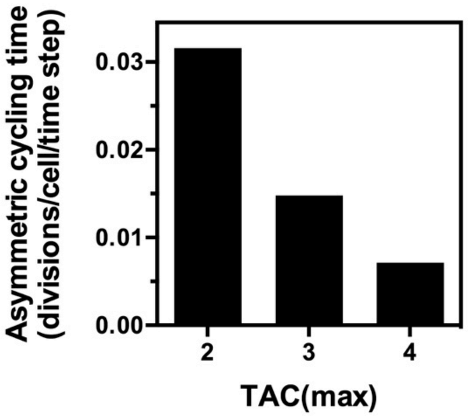

Supplementary Figure 2. LESCs cycle slower as the generational lifespan capacity of TACs increases. The time between asymmetric cell divisions of LESCs that can maintain corneal homeostasis is plotted for TAC(max) over the range of 2-4 generations.

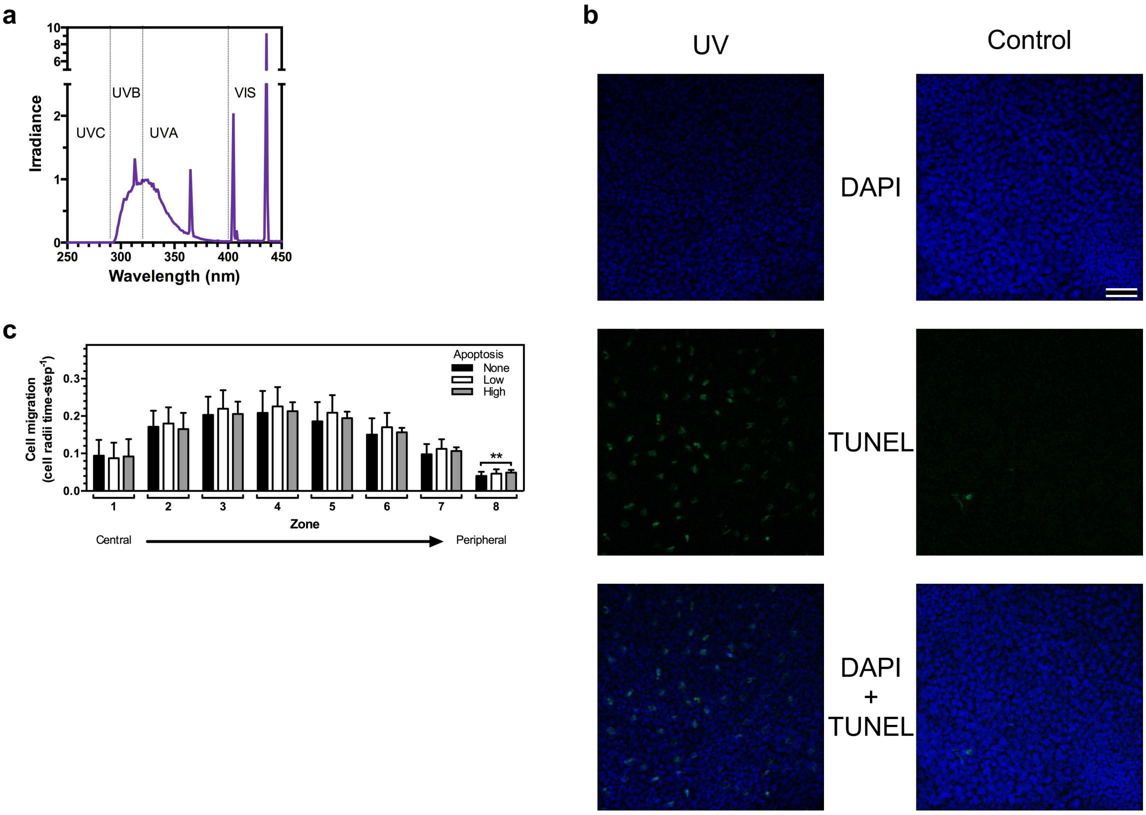

Supplementary Figure 3. UVR wounding of corneas. (**a**) The irradiance spectrum of the UVR source used to treat mouse corneas, as measured using an Optronics OL-754 spectroradiometer calibrated against standard lamps. The irradiance is shown relative to the intensity at 320nm. The boundaries of the UVA, UVB, UVC and visible (VIS) spectral regions are indicated. (**b**) TUNEL staining for apoptotic cells in UVR-treated corneal epithelium. The confocal microscopy image Z-stacks of full-thickness corneal epithelium were processed in Fiji software using the despeckle filter, rolling ball (50 pixel) background correction and maximum intensity projection. The individual channels for the nuclear counterstain, 4,6-diamidino-2-phenylindole dihydrochloride (DAPI), and TUNEL are shown above the composite images for a UVR treated eye (left) and the control eye (right) from the same mouse. Scale bar = 50µm. (**c**) Cell migration rates in UVR wounded simulated corneas return to normal levels soon after wounding ceases. The individual cell migration rates in the 8 annular zones of the simulated UVR treated and control corneas in Fig. 3f were calculated 85 time-steps later. The mean and SD of 25 simulations are shown. The only statistically significant difference in migration rates that remained was in the outermost zone, in which the cells of corneas subjected to a high level of damage (1.67% apoptosis per time-step) had a 23% higher migration rate than controls ($p=0.005$ by one-way ANOVA).

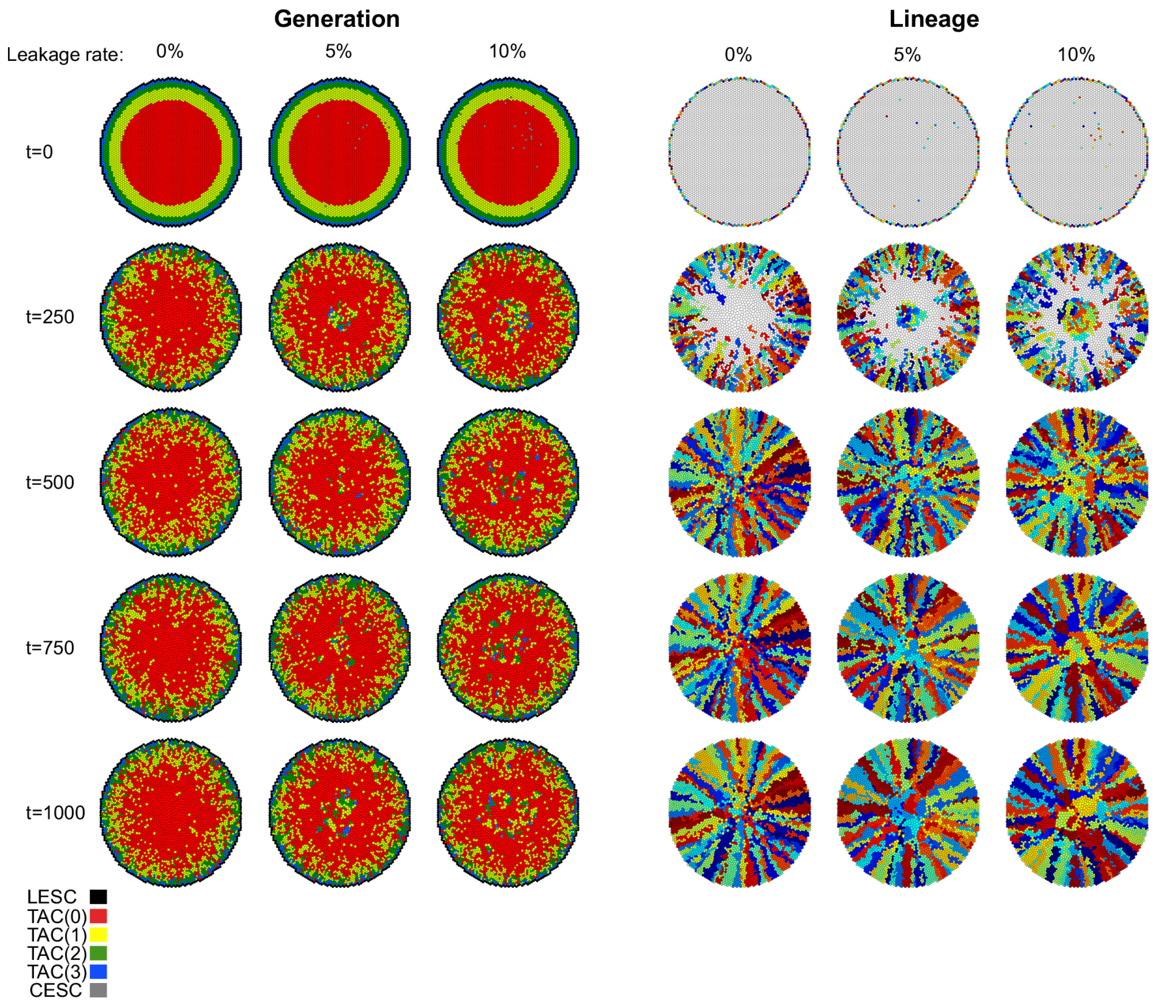

Supplementary Figure 4. **The effect of stem cell leakage on corneal pattern formation.** The generation maps (left) and corresponding clonal lineage maps (right) of the three corneas depicted in Fig. 4d-e, representative of 0%, 5% and 10% of LESC symmetric cell divisions resulting in a CESC. Time points at *t*=0, 250, 500, 750 and 1000 are shown.

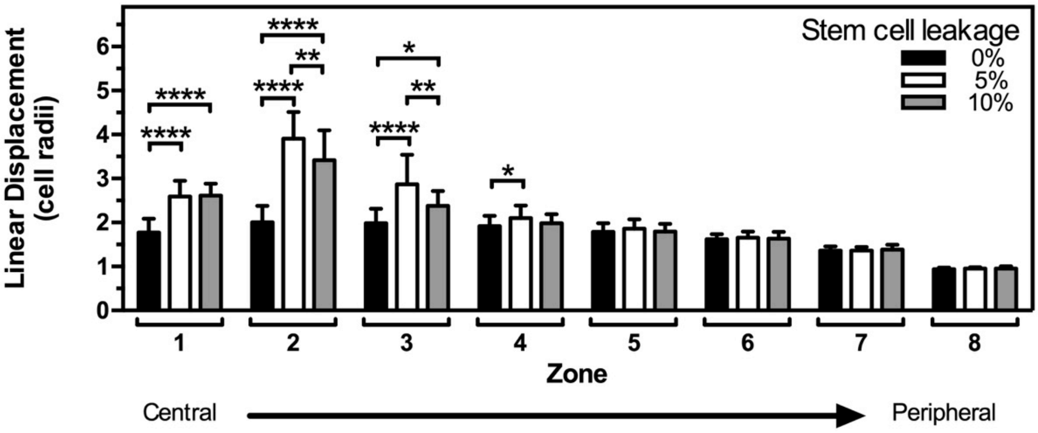

Supplementary Figure 5. Stem cell leakage increases disorganization near the center of the cornea. The LD was calculated for each zone depicted in Fig. 3e for all cell lineages in corneas in which there was no stem cell leakage from the limbus (0%) or 5% or 10% leakage. The mean±SD of the LD are shown for 25 simulations of each case. *p<0.05, **p<0.01, ****p<0.0001 by one-way ANOVA with Tukey's multiple comparisons test.

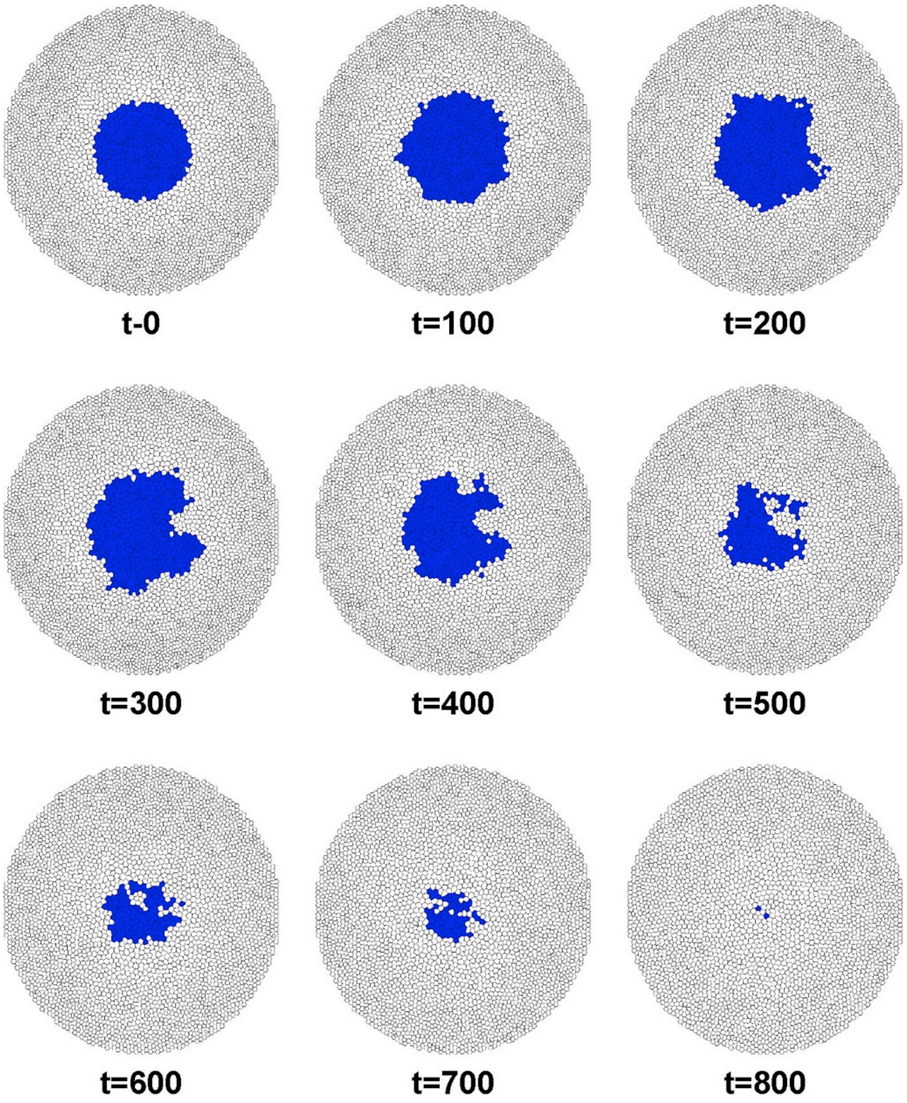

Supplementary Figure 6. CESCs do not form stable corneal epithelial cell lineages in the absence of stem cell leakage. Representative simulation of stem cells (shown in blue at the start of the simulation, t=0) transplanted into the central area of a recipient cornea (cells shown in white). All original transplanted cells and their progeny are shown in blue at 100 time unit intervals up to t=800. In these simulations, the LESCs of the recipient cornea produce TACs but do not leak stem cells into the cornea.

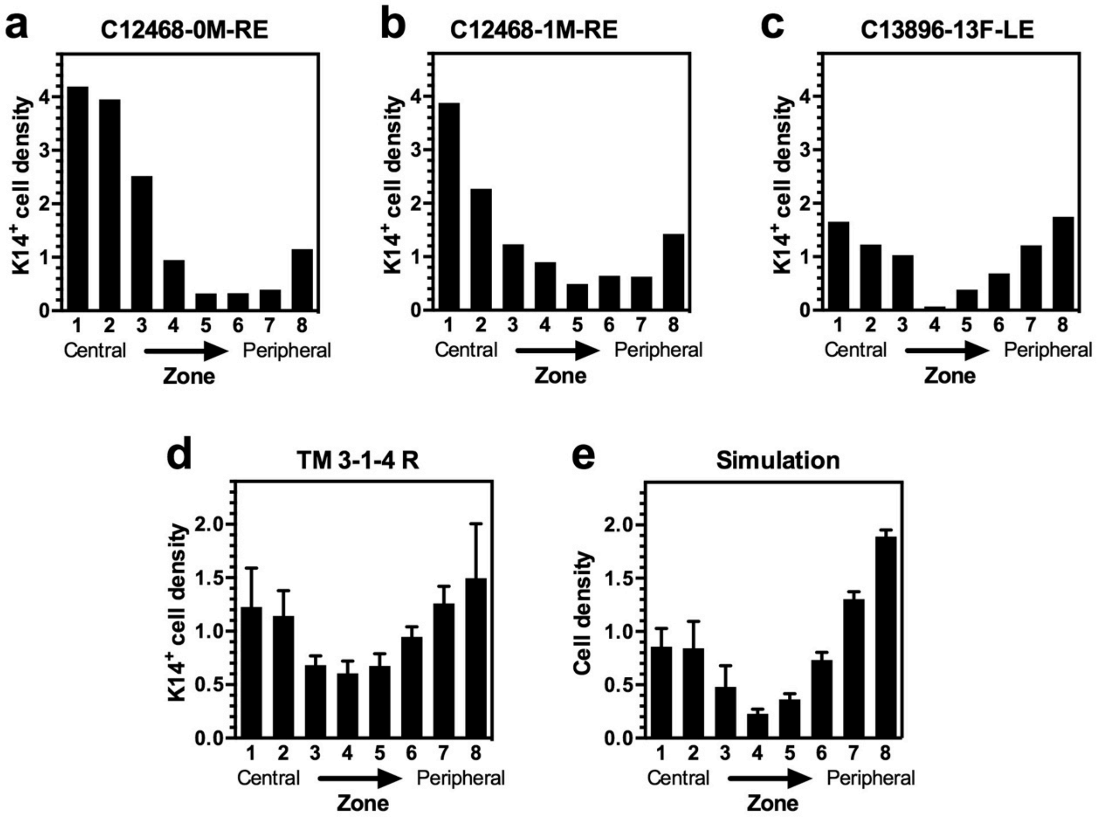

Supplementary Figure 7. Bimodal distribution of stem cells in corneas. Images of corneas were divided into 8 concentric zones, as in Fig. 5a, and the keratin-14 positivity of real mouse eyes (a-d), whose identification numbers are given above the graphs, or density of low generation number cells in simulations (e) were determined by intensity analysis using Fiji software. The real eyes (a-d) were from 4 independent mice injected with tamoxifen at 6 weeks of age and imaged 2 weeks later. The images analysed in a-c were obtained from the EYFP channel of living mice; those of d and e are the same as those in Fig. 5, b and d, respectively, and are included within the same figure here also for ease of comparison.

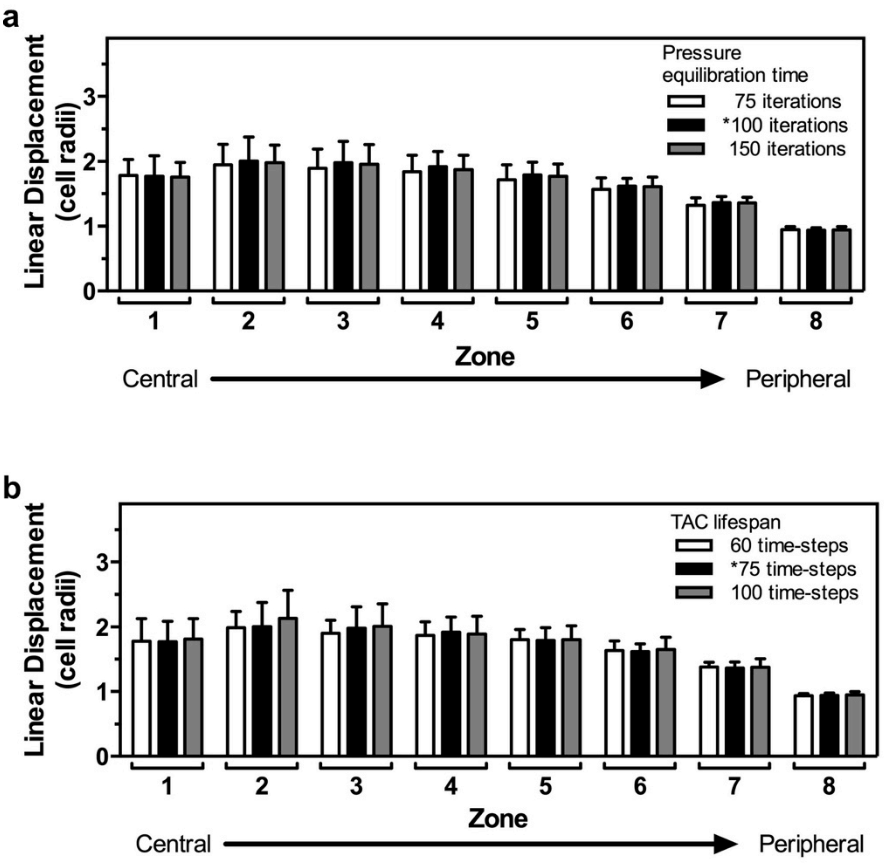

Supplementary Figure 8. Robustness of centripetal migration to variations in the pressure equilibration time and TAC lifespan in the simulation model. To determine whether centripetal migration in the simulation model is sensitive to variations in the pressure equilibration time in the movement rule or lifespan of TACs, the effects of increasing or decreasing these parameters from their usual levels were tested. The mean LD ± SD are shown for 25 simulations of each case. (**a**) During routine use of the simulation program, a maximum of 100 iterations of the simulation algorithm was allowed to occur at each time point to enable pressure equilibration to occur within the epithelial sheet. To determine whether the duration of pressure equilibration had a major effect on corneal pattern maintenance, the LD was also calculated for each zone depicted in Fig. 3 using 75 or 150 iterations per time point. (**b**) The lifespan of TACs was varied from the usual 75 time-steps to either 60 or 100 time-steps, as indicated, and the effect on centripetal migration was calculated and plotted as in (a).

Lineage	Number of cells	Mean direction (radians)	Mean linear displacement (cell radii)
#49	101	2.5102	1.12
#66	91	2.2209	1.40
#75	89	2.0802	1.96
#124	87	1.4921	1.64
#127	85	1.3844	1.23
#191	139	0.5495	1.50
#169	109	-0.7492	1.23
#53	106	-2.3481	1.68
#16	95	-2.8293	0.93
#7	96	-3.1082	1.08

Supplementary Table 1. Mean linear displacement of clones selected in Figure 1e. For each of the 10 clones plotted in Fig. 1e, the number of cells, the mean direction of the path from the LESC that gave rise to the clone and the mean linear displacement were calculated.

Parameter	*In vivo* parameter value	Simulation parameter value and comments
Shape of cornea	Circular dome	Flat circle. The ratio of central cells to peripheral cells will be somewhat lower in simulated corneas than in real corneas.
Size of cornea and basal corneal epithelial cell	The radius of a flat-mounted mouse cornea is ~100 cells and 1,500μm, giving the basal epithelial cells ~7.5μm radius[1]	The radius of the cornea is usually 30 cells, enabling 227 LESCs. This is large enough to maintain the complexity of the cornea and is the largest radius for computional tractability for routine simulations.
Predominant location of epithelial stem cells	The limbus[2]	The limbus
Replicative potential of TAC	"at least 2-3" cell divisions[3]	TAC(max) ranges from 2-4
Cell cycle time of TACs	3 days[3]; if 1/3 of these give rise to 2 TACs[4], then there are ~200h between symmetric TAC cell divisions	75 time-steps; thus, 1 time-step corresponds to ~2.7h
Spoke migration rate	11μm per day according to Di Girolamo et al[1] and this study. This corresponds to 1.4 cell radii day per day	The outcome of simulations at TAC(max)=3 is a spoke migration rate of 0.14 cell radii per time-step (Fig. 3), which is 1.2 cell radii per day
UVB-induced apoptosis	Occurs over 2 days, peaking at 3.7% of basal cells (this study)	Either 0.67% (low) or 1.67% (high) per time step for 25 time steps.

Supplementary Table 2. Key biological parameters observed *in vivo* and either chosen as input for the simulation model or produced by the model as output.

Supplementary Methods

ODD Protocol of the mathematical model

1. Purpose

The model is used to investigate the key mechanisms of corneal epithelial maintenance. Specifically, it can be used to investigate the roles of stem cell location, population pressure, cell longevity, replicative capacity, growth rate and migration on the clonal lineage structure of the cornea.

2. Entities, state variables, and scales

The model space is a circular region representing the cornea and of adjacent limbus consisting of approximately 4,000 cells within the basal layer. Each cell has the following attributes, or state variables:

1. Position: The position of a cell is given determined by a single pair of co-ordinates, as we ignore the curvature of the basal layer of the cornea. Collectively, the set of cell positions is used to determine the edges and vertices of cells, using geometric structures known as Voronoi diagrams. We assume that the limbus consists exclusively of stem cells, which are all within a single cell rim a fixed region away from the center of the corneal region.
2. Lineage identification: Each stem cell is endowed with a distinct lineage identification code, and all future progeny of that stem cell in the cornea share the same cell lineage identification code.
3. Age: Cells are attributed with a current age. Processes such as time to cell death, symmetric cell division and differentiation off the basal layer are assumed to occur approximately periodically.
4. Type: Each cell is characterized by a type, reflecting either the phenotypic behavior of cells in the cornea, or they are used to maintain the model. Cell types are: Ghosts, whose position is exterior to the circular region that represents the cornea, and are designed to play no active part in corneal dynamics, but are necessary to maintain the spatial structure of the model; epithelial stem cell (ESC) types that represent the phenotypic properties of stem cells found either in the limbus (LESC) or in the cornea (CESC); and transit amplifying cell (TAC) types within the cornea. CESCs behave like LESCs with respect to lifespan and replication, but like TACs with respect to motility and spatial assignment of daughter cells following cell division. Cell types ESC and TAC differ in the following ways:

- Lifespan: The lifespan of ESCs are normally distributed with a mean and standard deviation of 750 and 25 time steps, respectively. The lifespan of TACs are normally distributed with a mean and standard deviation of 75 and 25 time steps, respectively.
- Replicative capacity: ESCs are assumed to have a replicative capacity that is much greater than the time scale represented by the model, and are therefore characterized by limitless replicative potential for both symmetric and asymmetric proliferation in the model. ESCs are assumed to replicate symmetrically, producing two ESCs, or asymmetrically, producing an ESC and a TAC. TACs can divide asymmetrically to

produce another TAC and a terminally differentiated cell (TDC). TDCs do not contribute to the basal layer and are not depicted in simulations. TACs have a limited number of rounds of symmetric cell division, known as TAC(max), after which they divide symmetrically to produce two TDCs, both of which do not contribute to the basal layer.

- Replicative frequency: Replication rates for ESCs and TACs are chosen to maintain corneal equilibrium over time.

3. Process overview and scheduling

At each time step, the following processes occur in this order:

1. Cell motility
2. Render and record the state of the simulation
3. Age cells
4. Cell division of ESCs
5. Differentiation off the basal layer
6. Cell division of TACs

4. Design concepts

1. Basic principles: We make the following assumptions:
   a. the cornea is in a state of homeostasis, conforming with the X, Y, Z hypothesis[5]
   b. the cell lineage pathways in the cornea are ESC-TAC-TDC
   c. there is a fixed population of ESCs in the limbus
   d. the cells in the cornea are motile
   e. the population pressure is related to the density of neighboring cells
   f. cells respond to pressure gradients by moving from higher pressure to lower pressure areas.
2. Emergence: the spatial structures of cell lineages in the cornea emerge through passive motility rules, driven by a cellular spring network.
3. Adaptation: cells in the cornea move in response to the pressure exerted by their immediate neighbors and either replicate or die in response to reaching their lifespan; LESCs respond to the death of a neighbor by replicating themselves; these responses do not adapt over time or in response to position.
4. Objectives: the cells do not aim to meet any objectives.
5. Learning: individual cells do not change their adaptive traits over time in response to experience.
6. Prediction: there are no adaptive traits in the model, so the agents in the model do not make any predictions.
7. Sensing: cells can sense the pressure exerted by their neighbors and whether they are located in the limbus or the cornea.
8. Interaction: cells respond to the local configurations of other cells by moving in response to a pressure gradient or, in the case of LESCs, replicating in response to the death of a neighbor.
9. Stochasticity: the lifespans of cells are normally distributed for both ESCs and TACs. The other source of stochasticity in the model occurs when a neighboring LESC is

selected to undergo symmetric cell division in response to the removal (death) of a LESC.

10. Collectives: there is no pre-determined collective behavior in the model.
11. Observation: position, lineage identification code, age and proliferative capacity is recorded for all cells in the model at every time step.

5. Initialization:

Cell positions are initially chosen so that the Voronoi diagram generated is a regular hexagonal grid (the distance between the positions of neighboring cells is a constant, s, known as the idealized cell diameter). All ESCs are given a random age (uniformly distributed from 0 to their lifespan), and a unique cell lineage identification code. Initially, all TACs are not assigned a lineage identification code. TACs derived from ESCs after initialization inherit the lineage identification code of their parent cell. Cell types are chosen such that LESCs have only two adjacent LESC neighbors, and that TACs that have less proliferative capacity are placed towards the center of the cornea. This placement has been validated through simulations where the proliferative capacity of TACs had no spatial relationship, and the simulations were run until homeostasis was reached. At that point, TACs near the center had less proliferative capacity than cells near the limbus.

6. Input data:

There are no external input data that alter the model during the simulations.

7. Sub-models:

Cell motility: Cell motility in response to pressure: the cell positions of all TACs are updated through a cell movement rule, which is applied until cells are deemed to have reached a fair approximation of their final position i.e. when further application of the movement rule does not move cell positions over a particular threshold, and cells neighboring cells are sufficiently close to each other. At each time step that the movement rule is applied, the set of Voronoi neighbors (found through Delaunay Triangulation) is found, and cell movement arising from pressure between a TAC a neighboring cell is proportional to the d_p-s, where d_p represents the distance between the cell neighbors, and s represents a single cell diameter. The total movement of a cell is the sum of all movements arising from each pair of neighbors, and this is done synchronously. The positions of both LESCs and Ghosts are fixed.

1. Cell motility: Cell positions are updated through a cell movement rule that is applied repeatedly, until cell positions are deemed to have reached a fair approximation of their final position i.e. when further application of the movement rule does not move cell positions more than $s/2$, and the distance between the cell positions of neighboring cells is less than $4s$. The cell movement rule changes p_i, the position of a non-LESC cell, i, by Equation 1:

$$\Delta p_i = \frac{1}{2\sqrt{3}} \sum_j \frac{w_{i,j}(p_i - p_j)(s - |p_i - p_j|)}{|p_i - p_j|}$$

where j represent the cells that neighbor cell, i, and $w_{i,j}$ is the length of the shared cell wall between cells i and j, and leaves the position of LESCs unperturbed. Each time the cell movement rule is applied, the set of Voronoi neighbors (found through Delaunay Triangulation) is recalculated.

2. Render and record the state of the simulation: All data and figures are collected during this point in the process. Cells with a particular replicative capacity remaining are color-coded in generational maps, and cells that share the same lineage identification code are assigned to a specific color, unique to that identification code in the clonal lineage maps.
3. Age cells: All cells are assumed to age synchronously.
4. Symmetric and asymmetric cell division of ESCs:
   - LESCs that have reached their lifespan are removed, and are synchronously replaced by symmetric proliferation of a neighboring LESC, which is chosen at random. The cell lineage identification of the new cell matches that of the mother cell, and the lifespan of the new cell is chosen randomly from $N(750, 25)$.
   - In the representations where LESCs can give rise to CESCs, the other neighboring LESC gives rise to a CESC, with probability p. In this case, the CESC shares the lineage identification of the mother cell, and its position is $0.5s$ closer to the center of the cornea than the mother cell, and the lifespan of the CESC is chosen randomly from $N(750, 25)$.
   - LESCs give rise to TAC(max)s at a rate designed to maintain corneal equilibrium. Newly proliferated TACs share the lineage identification of the mother cell, its position is 0.5s closer to the center of the cornea than the mother cell, and the lifespan of the TAC(max) is chosen randomly from $N(75, 25)$. The rate proliferation rate r_{lt} of LESCs to TACs is given by Equation 2:

   $$r_{lt} = \frac{3679}{227 * 75 * (2^{max+1} - 1)}$$

   as 227 is the number of LESCs, which enclose an average 3769 interior cells, and 75 is the average age of TACs.
5. Cells differentiation off the basal layer: TACs that have reached their lifespan and exhausted their replicative potential die by becoming TDCs in the suprabasal layers of the epithelium, and are removed from the model.
6. Symmetric division of a TAC with x rounds of cell division remaining produces two daughter TACs, both having $x-1$ potential rounds of cell division remaining. Both these daughter cells share the lineage identification code as the mother cell, and are placed within 0.5s of the position of the mother cell, with no other predetermined spatial assignment.

Supplementary References

1 Di Girolamo, N. *et al.* Tracing the fate of limbal epithelial progenitor cells in the murine cornea. *Stem Cells* **33**, 157-169 (2015).

2 Davanger, M. & Evensen, A. Role of the pericorneal papillary structure in renewal of corneal epithelium. *Nature* **229**, 560-561 (1971).

3 Lehrer, M. S., Sun, T. T. & Lavker, R. M. Strategies of epithelial repair: modulation of stem cell and transit amplifying cell proliferation. *J. Cell Sci.* **111**, 2867-2875 (1998).

4 Lamprecht, J. Symmetric and asymmetric cell division in rat corneal epithelium. *Cell Tissue Kinet.* **23**, 203-216 (1990).

5 Thoft, R. A. & Friend, J. The X, Y, Z hypothesis of corneal epithelial maintenance. *Invest. Ophthalmol. Vis. Sci.* **24**, 1442-1443 (1983).
